# Proteomic analysis of plasma after branched chain enriched mixture supplementation in mice

**DOI:** 10.1186/1550-2783-10-19

**Published:** 2013-04-03

**Authors:** Lorenza Brocca, Anna Mascaro, Giuseppe D’Antona

**Affiliations:** 1 Department of Molecular Medicine, University of Pavia, Pavia, Italy; 2 LUSAMMR, Laboratory for Motor Activities in Rare Diseases, Sport Medicine Centre Voghera, Voghera, Italy

**Keywords:** Amino acids, Proteome, Dietary supplements

## Abstract

**Background:**

Branched chain amino acid (BCAA) supplementation is a recently identified strategy to promote longevity in mice. A proteomic approach was used to identify proteins which are differentially expressed in the sera of mice following supplementation with selected branched chain amino acid enriched mixture (BCAAem).

**Findings:**

12 male mice (C57Bl6, 9 months-old) were randomly assigned to unsupplemented (Control, n = 6) and supplemented (BCAA, n = 6, 0.1 mg/gr/day in drink water for 4 weeks). At the end of treatment total plasma samples from Control and BCAAem mice were separated by two-dimensional gel electrophoresis (2-DE). After staining, the gels were imaged and differential protein expression patterns were interrogated using image analysis software. Spots showing a different expression level were identified through a comparison with 2D maps found in databases officially recognized (ExPASy).

Master gels of Control and BCAA mice exhibited slightly different 2-DE patterns as only 10 spots out of 500 appeared differentially expressed: 8 were upregulated (corresponding to Apolipoprotein A-I (APOA1), Complement factor B, Complement C3, Immunoglobulin light chain) and 2 appeared downregulated (Alpha-1-antitrypsin and unknown).

**Conclusions:**

Supplementation with BCAAem in mice results in a slight perturbation of the host serum proteome. Of particular interest is the increased Apolipoprotein A-I (APOAI) following treatment.

## Background

In ageing common metabolic, inflammatory, cardiovascular and neurodegenerative diseases, ultimately reduce healthspan and lifespan.

Regardless of the mechanism, a common feature of aging-related diseases is the involvement of metabolic systems in general, and the mitochondria in particular [1].

We have recently demonstrated that supplementation of aged mice with a branched-chain amino acid-enriched mixture (BCAAem) promotes mitochondrial biogenesis and function, with a reduced radical oxygen species (ROS) production and extension of mean survival [2]. All the BCAAem-mediated effects appeared to be considerably enhanced by combined resistance exercise training and strongly attenuated in endothelial nitric oxide synthase null-mutant mice (eNOS^−/−^) or after rapamycin, an inhibitor of mammalian target of rapamycin (mTOR) pathway. Although a direct metabolic effect of BCAAem on skeletal muscles contributes to the overall change in mitochondrial biogenesis and function and antioxidant activity [2], an indirect tissue effect mediated or sustained by circulating factors may contribute to the observed effects on survival or, simply, may represent footprint biomarkers of the nutritional strategy. This concern might also be considered in order to clarify the mechanisms underlying the known beneficial effect of BCAA supplementation before and after exercise mainly consisting in decreased exercise-induced muscle damage and promoted muscle protein synthesis [3]. Indeed initial reports highlight the effects of BCAA enriched mixtures supplementation on the pattern of circulating factors such as cytokines [4] and hormones (i.e. GH) following exercise in humans [5].

Here we used plasma proteomics to investigate whether dietary supplementation with BCAAem would impact on the plasma protein profile thus defining a plasma biomarker fingerprint of supplementation in adult sedentary mice.

## Methods

12 male mice (F2 Hybrid B6.129S2 obtained from crossing C57BL/6 J and 129S1/SvImJ mice, 9 month at the beginning of treatment) (The Jackson Laboratory), housed one per cage and maintained at 20°C, 12 h/12 h day/night cycles, were treated according to the EU guidelines and with the approval of the Institutional Ethical Committee. Animals were given unrestricted access to a standard diet (4.3 kcal% fat, 18.8 kcal% protein, 76.9 kcal% carbohydrate, Laboratorio Dottori Piccioni) and were randomly assigned to two groups: unsupplemented (Ct, n = 6) and supplemented (BCAA, 0.1 gr/kg/day in drinking water, n = 6). Consumption of food and water was monitored along the treatment and appeared not statistical different between groups. (Ct, 3.1 ± 0.01 g/day and 6.5 ± 1.0 ml/day, n = 6; BCAA, 3.3 ± 0.03 g/day and 6.0 ± 1.2 ml/day, n = 6 respectively p > 0.05). The amino acid supplement BCAAem (composition: 31.25% leucine, 16.25% lysine, 15.52% valine, 15.52% isoleucine, 8.75% threonine, 3.75% cysteine, 3.75% histidine, 2.6% phenylalanine, 1.25% methionine, 0.75% tyrosine, 0.5% tryptophan) was administered with a daily dose of 0.1 gr/kg body weight dissolved in tap water on basis of the previously monitored daily drinking (average drinking 6.65 ± 1.5 ml/day, n = 12).

At the end of treatment in the late morning and after at least 4 h fasting, mice were weighted (Ct, 30 ± 1 g n = 6; BCAA 29 ± 1.2 g n = 6, p > 0.05) and a blood sample (around 400 μL) was withdrawn from the retro orbital sinus of each mouse under slight ether anesthesia.

The samples were centrifuged at 8000 g for 15 min in order to separate the serum fractions which were frozen in liquid nitrogen and maintained at −80°C for subsequent analysis.

### Two-dimensional electrophoresis analysis

Protein concentration of each sample were determine using the *DC* Protein Assay (by Bio-Rad), a colorimetric assay based on the method of Lowry [6].

100 μg of protein for each sample (Ct and BCAA) were precipitated in 8 volumes of acetone and then resuspended in a 2D lysis buffer (8 M urea, 2 M thiourea, 4% Chaps, 65 mM DTT and 40 mM Tris base). All Ct samples were combined to create a Ct sample mix and the same was done for samples BCAA. 150 μg of protein from each sample mix were used to perform the 2D-electrophoresis analysis. Isoelectrofocusing was carried out with the IPGphor system (Ettan IPGphor isoelectric focusing system, GE Healtcare) using IPG gel strips pH 3–11 NL, 13 cm long. Gel strips were rehydrated for 14 hours, at 30 V and 20°C, in 250 μl of reswelling buffer (8 M urea, 2 M thiourea, 2% Chaps, 0.1% tergitol NP7, Sigma) and focused at 20000 V/h at 20°C. After they were incubated 10 min in equilibration buffer (50 mM Tris pH 6.8, 6 M urea, 30% glycerol, 2% SDS, 3% iodoacetamide) before being applied on 15% SDS-Page gel without staking gel. The separation of protein spots was performed at 80 V for 17 h at room temperature.

After having been fixed for 2 hours in a fix solution (40% ethanol, 10% acetic acid), the 2D gels were stained with fluorescent staining (Flamingo™ fluorescent gel stain, Bio-Rad) for 3 hours and destained in 0.1% Tween 20 solution for 10 min.

For each mix samples we obtained three different gels visualized by Typhoon laser scanner (GE Healtcare) and then analyzed with Platinum software (GE Healtcare). The software compared BCAA with Ct group by choosing a master gel used for the automatic matching of spots in other 2D-gels. At the end the analysis we obtained for each spot the normalized volume representing the protein amount. Then we averaged the volumes of the corresponding spots in three replicate gels getting spots that statistically changed (p < 0.05).

Finally we compared our proteomic maps with those published on specific databases (ExPASy) in order to identify differentially expressed spots.

### Statistical analysis

Statistical analysis was performed with GraphPad Prism® 5.02 software (GraphPad Software, San Diego, CA). Results are expressed as means ± standard deviation of the mean (SD). Statistical significance was calculated using unpaired Student’s *t*-test. Statistical significance was set to p < 0.05.

## Results

**Figure 1:**
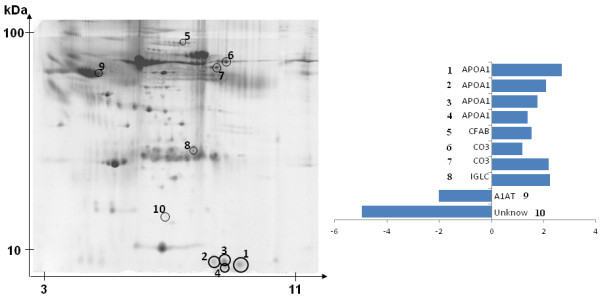
**Example of typical 2-DE gel image of plasma proteins extract.** Left, Changed spots circled and numbered. Right, Identified proteins and fold changes. APO A-I, Apolipoprotein A-I; CFAB, Complement Factor B; IGCL, Immunoglobulin light chain; A1AT, Alpha-1-antitrypsin.

Representative 2-DE gels for Ct and BCAA are reported in Figure 1 and identity and fold changes of identified plasma proteins are reported in Table 1. By matching 2D gels from Ct and BCAA around 500 common spots were analyzed whereas only 10 spots appeared differentially expressed. Among them 8 appeared upregulated and identified as Apolipoprotein A-I (APOAI), Complement factor B, Complement C3, Immunoglobulin light chain and 2 appeared downregulated identified as Alpha-1-antitrypsin and unknown.

**Table 1:** Identification of changed plasma protein following BCAAem supplementation by ExPASy

	**Protein name**	**Protein name**	**Accession number**	**Fold change**	**Physiological function**
1	Apolipoprotein A-I	APOAI	Q00623	2.70	Partecipates in RTC from tissues to liver
2	Apolipoprotein A-I	APOAI	Q00623	2.10	Partecipates in RTC from tissues to liver
3	Apolipoprotein A-I	APOAI	Q00623	1.80	Partecipates in RTC from tissues to liver
4	Apolipoprotein A-I	APOAI	Q00623	1.38	Partecipates in RTC from tissues to liver
5	Complement factor B	CFAB	P04186	1.54	Is part of the alternate pathway of the complement system
6	Complement C3	CO3	P01027	1.19	Plays a central role in the activation of the complement system
7	Complement C3	CO3	P01027	2.20	Plays a central role in the activation of the complement system
8	Immunoglobulin light chain	IGCL	Q925S9	2.24	
9	Alpha-1-antitrypsin	A1AT	P07758	- 2.03	Inhibitor of serine proteases
					Acute phase response
10	Unknow			−4.97	

## Conclusions

As far as we know this is the first available proteomic analysis of the plasma proteins expression profile after BCAA enriched mixture supplementation in mice.

Results showed that 1) a limited number of proteins changed expression in the plasma of supplemented in comparison with control unsupplemented animals; 2) four spots showed significant quantitative fold changes and were identified as Apolipoprotein A-I.

In our study the oral supplementation with BCAAem for four weeks was associated with a minor change of the 2-DE pattern profile as only 10 spots out of 500 appeared differentially expressed between supplemented and unsupplemented mice. In particular the upregulated spots were identified as Apolipoprotein A-I, Complement factor B, Complement C3, Immunoglobulin light chain whereas the downregulated spots were Alpha-1-antitrypsin and an unidentified protein.

Apolipoprotein A-I is a major protein component of high density lipoprotein (HDL) in the plasma and participates to the reverse cholesterol transport (RCT) from tissues to liver where it can be excreted directly into the bile or metabolized into bile salts before excretion [7,8]. Lipid-poor Apo A-I/HDL are known to act as acceptors for cellular lipids, and lipid efflux from cells can be mediated via cell surface proteins (ABCA1, ABCG1 and SR-BI) [9]. RCT represents the foremost mechanism underlying the anti-atherogenic effects of Apo A-I. Apart from its participation to the RTC HDL/Apo A-I might exert their anti-atherogenic effects through several other mechanisms. For example, it has been demonstrated that HDL/Apo A-I have anti-inflammatory activity [10] being capable to reduce oxidized lipids and its inflammatory effects [11,12].

In experimental studies using atherosclerosis-susceptible mice (inbred C57BL/6, used in the present study), it was observed that transgenic overexpression of human ApoA-I significantly protected from development of early atherosclerotic lesions [13]. Similarly, overexpression of human ApoA-I in apoE-deficient transgenic mice suppressed early atherosclerotic lesions [14]. Furthermore, knocking out apoA-I resulted in an accelerated atherosclerosis development in several animal models (i.e. the human apoB-transgenic female mice; the LDL receptor-deficient; the LDL receptor/apoE-deficient mice) [15,16].

Taking into account that increasing ApoA-I production is now considered a target for coronary heart disease (CHD) risk reduction, beside pharmacological agents, several studies have focused on nutritional compounds affecting serum apoA-I concentration. For instance it has been found that, saturated fatty acids (SAFAs) and cis-monounsaturated fatty acids (cis-MUFAs), lecithin (consisting of three phospholipids; phosphatidylcholine (PC), phosphatidylethanolamine (PE), and phosphatidylinositol (PI)) and moderate amounts of ethanol [17] increase serum ApoA-I concentrations [18] but the mechanisms underlying these changes remain to be fully elucidated. Beside the energy-delivering nutrients diverse micronutrients, such as minerals (e.g. zinc, magnesium, and vanadate) and vitamins (e.g., vitamin C, vitamin D, vitamin E, and vitamin A, vitamin B3), might also enhance ApoA-I synthesis [19,20]. Anyhow whether these findings also hold for the in vivo situation remains to be confirmed [21]. In our study we describe, for the first time, an increased Apo A-I plasma concentration following BCAA enriched mixture supplementation in the wild type mouse. The likely role of essential amino acids in Apo A-I synthesis deserves future investigations.

In this study, we observed an increase in Complement C3 (CO3) and Complement Factor B (CFB) plasma proteins. CO3 plays a central role in the complement system activation. Its processing by C3 convertase is the central reaction in both classical and alternative complement pathways. After activation C3b can bind covalently via its reactive thioester to cell surface carbohydrates or immune aggregates [22]. Elevated C3 concentrations were associated with increased risk of impaired insulin sensitivity, insulin resistance, abdominal obesity and low HDL cholesterol compared to low C3 concentrations. Increased CHD risk conferred by elevated C3 concentrations is further accentuated among high dietary fat consumers and monounsaturated fat [23].

CFB is a fundamental component of the alternative complement pathway. Following the activation of alternative pathway factor B is cleaved by complement factor D into 2 fragments of different molecular weight, Ba (noncatalytic chain) and Bb (catalytic chain). Both of these fragments express a variety of biological functions. In particular Bb is a serine protease that combines with complement factor 3b to generate the C3 or C5 convertase. Bb is involved in the proliferation of preactivated B lymphocytes, while Ba inhibits their proliferation.

Factor B hyperconsumption and increased catabolism, concomitant with factor B fragment production, occurs in a wide variety of diseases, including gram-negative sepsis, autoimmune diseases and burns [24] whereas very few data are reported on the effects of dietary supplementations on CFB plasma levels [25,26]. An increased CFB concentration could enhance the immune response of the alternative pathway, by providing more factors B to be spun to generate more C3-convertase thus increasing the amount of its secondary reactions described above.

Although the significance of the observed changes and the underlying mechanisms deserve future investigations, the evidence of a contemporaneous increase of Apo A-I and Complement proteins allow us to speculate about a protective role of increased HDL following supplementation. In fact, *in vitro* studies indicate that HDL blocks the assembly of the terminal complement attack complex on endothelial cells [27]. Indeed the observed decrease in Alpha-1-antitrypsin (A1AT) a serine proteases inhibitor related to acute phase response [28] is probably a sign of the improvement in HDL protective capabilities sustained by BCAAem supplementation.

Finally in our analysis we found an increase in Immunoglobulin light chain (IgLC) levels. Studies have shown that IgLC can bind to mast cells facilitating their activation [29] thus contributing to the development of inflammatory disease. Furthermore excess of IgLC may modulate the apoptotic cell death of neutrophils thus contributing to increased susceptibility to bacterial infections in presence of renal failure [30,31]. Considering that only one spot identified as IgLC appeared to be increased following supplementation and that no signs of renal dysfunction have been detected following long-term BCAAem supplementation [32], quantitative and qualitative significance of the change observed in our study remains to be elucidated.

### Limitations of the study

Our study has limitations. First our results are to be considered preliminary as only an age, 9 months corresponding to adulthood in mice, has been analyzed. Second, the identification of proteins was based on available proteome database in the mouse (ExPASy) and not on mass spectrometry. Anyhow we reckon that the latter limitation is not a major bias as, to date, available databases on proteome of mouse plasma are highly reliable. Furthermore a direct translation of results to human beings in unlikely as the daily dose usually adopted in mice (0.1gr/gr/day) are around ten fold those suggested in humans (0.1gr/kg/day), as in mice dose correction is made for the higher basal metabolism [33].

Notwithstanding these limitations, results from our study opens up a new avenue of research, aimed to identify the individual contributions of these molecular markers to the effects of BCAA enriched mixtures supplementations in mammals.

## Competing interests

The authors declare non conflicts of interests.

## Authors’ contributions

LB participated in statistical analysis and manuscript preparation, AM participated in data collection, statistical analysis. GD served as the principal investigator and contributed to study design, data collection, and manuscript preparation. All authors read and approved the final manuscript.
